# TCRP1 promotes NIH/3T3 cell transformation by over-activating PDK1 and AKT1

**DOI:** 10.1038/oncsis.2017.18

**Published:** 2017-04-24

**Authors:** C Wang, H Liu, Q Qiu, Z Zhang, Y Gu, Z He

**Affiliations:** 1 Cancer Hospital and Cancer Research Institute, Guangzhou Medical University, Guangzhou, China; 2 Cancer Research Institute, University of South China, Hengyang, China

## Abstract

Tongue cancer resistance-related protein 1 (TCRP1) gene was first cloned from the multidrug resistance tongue cancer cell (Tca8113/pingyangmycin) in our lab. Our precious studies demonstrated that TCRP1 was involving in chemotherapy and radiotherapy resistance of tongue cancer cells, lung cancer cells and ovarian cancer cells. In this study, we showed that TCRP1 overexpression promotes cell transformation and tumorigenesis through hyperphosphorylation of the oncogenic kinase 3-phosphoinositide-dependent protein kinase-1 (PDK1) and AKT1, whereas inhibition of PDK1 by OSU-03012 or PDK1 small interfering RNA reversed TCRP1-mediated cell transformation. Importantly, TCRP1 was able to directly interact with PDK1, and 93–107 amino-acid and 109–124 amino-acid sites of TCRP1 were the common binding domain of PDK1. Moreover, in line with its oncogenic activity, we found that TCRP1 is often overexpressed in human in lung cancer, glioma, ovarian cancer, thyroid cancer, nasopharyngeal carcinoma, pancreatic cancer, stomach cancer and tongue carcinoma tissues. Spearman correlation analysis showed that the expression of TCRP1 has a positive correlation with p-PDK1, as well as p-AKT1 in lung cancer and gliomas tissues. Thus, TCRP1 may be a candidate as human oncoprotein that promotes cancer development by activation of PDK1/AKT1 signaling.

## Introduction

Cancer is a complex and diverse set of diseases related to the unharnessed growth, enhanced survival and invasion of cells. Although there are various means to achieve cellular transformation, a limited number of elements can suffice to transform many different human cell types, suggesting that a discreet yet common set of pathways mediate this process.^1^ The phosphoinositide 3-kinase (PI3K)-AKT pathway is one of the most commonly deregulated signaling pathways in human cancers.^2^ Genetic lesions that result in increased PI3K pathway signaling, such as activating mutations of PIK3CA or inactivation of phosphatase and tensin homolog deleted on chromosome 10 (PTEN), are a hallmark of a staggering number of malignancies emanating from many tissue types.^3^ The 3-phosphoinositide-dependent protein kinase-1 (PDK1) is known to be activated as a result of the accumulation of the PI3K product phosphatidylinositol-3,4,5-trisphosphate (PIP3), and considered an important component of the PI3K pathway.^4^ PDK1 serves as a master regulator of the AGC family of protein kinases, which includes AKT, S6K1, SGK and PKC, and thus having multiple roles in various physiologic processes such as growth, proliferation and survival.^5^ Given the central role of PI3K/PDK1/Akt signaling as an oncogenic driver of tumorigenesis, more complete characterization of the interconnections between PI3K pathway components and different oncogenic pathways is necessary to identify underlying mechanisms of tumorigenesis that may be exploited therapeutically.

Tongue cancer resistance-associated protein 1 (TCRP1) was cloned by our lab from the tongue cancer multidrug resistance cell line Tca8113/pingyangmycin, which showed resistance to pingyangmycin, cisplatin, pirarubicin, paclitaxel and adriamycin.^6^ TCRP1 gene is located on chromosome 11, q13.4, contains 8 exons. Molecular weight of TCRP1 is about 25 kDa, located in the cytoplasm, and does not have a transmembrane structure and secreted signal peptide.^7^ Our previous studies have shown that TCRP1 was involved in chemotherapy and radiotherapy resistance of tongue cancer cells, lung cancer cells and ovarian cancer cells.^8, 9, 10^ Co-immunoprecipitation experiments and glutathione *S*-transferase-pull down assays showed that TCRP1 directly interacts with Akt, and then activates AKT/nuclear factor-κB/mammalian target of rapamycin signaling, which in turn, promotes cell proliferation and inhibits cells apoptosis.^8, 10^ However, the function of TCRP1 in tumor initiation and development has not been well characterized.

In this study, we found that TCRP1 participates in cell transformation and cancer development through activation of PI3K/PDK1/AKT1 signaling.

## Results

### TCRP1 overexpression promotes cell transformation of NIH/3T3 cells

**Figure 1:**
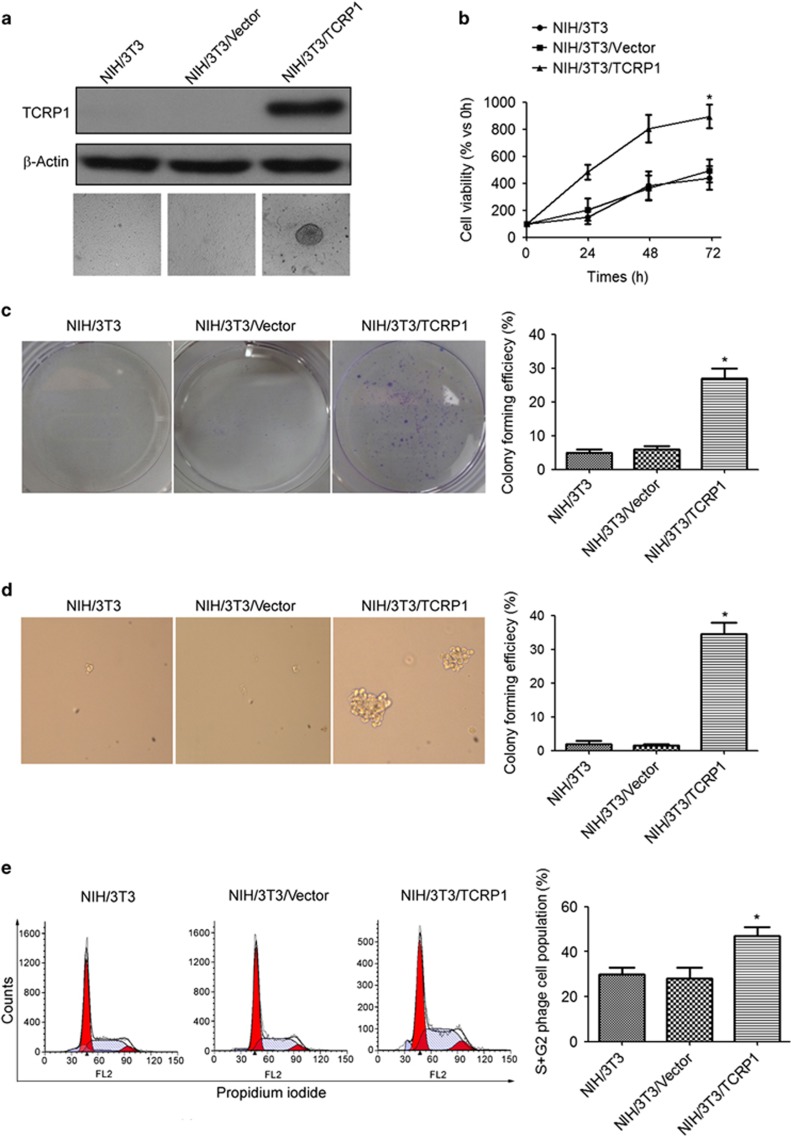
TCRP1 overexpression promotes cell transformation of NIH/3T3 cells. NIH/3T3 cells were transfected with TCRP1-overexpressing vector, (**a**) TCRP1 expression was analyzed by using the western blot method. β-Actin was used as an inner control. (**b**) Cell viability was measured by MTS assay. (**c**, **d**) Clonogenic capacity was measured by plate cloning assay (**c**) and soft agar cloning assay (**d**). (**e**) Cells were fixed in ethanol, and stained with propidium iodide, and then DNA contents were determined by flow cytometry. The percentage of cells with G1, S and G2 was calculated using Flowjo analysis software. Experiments were repeated three times. *P <0.05.

We first asked whether TCRP1 overexpression could promote transformation of NIH/3T3 cells. To this aim, pLEX-based constructs encoding TCRP1 or with empty vector (CT) were transfected into NIH/3T3 cells (Figure 1a). Our results showed that overexpression of TCRP1 significantly increased cell proliferation (Figure 1b). Plate cloning and soft agar cloning assay also showed that clonogenic capacity of NIH/3T3/TCRP1 cells was significantly enhanced compared with the control group (Figures 1c and d). Moreover, flow cytometric analysis showed that the cell numbers in the S phase and G2 phase were significantly increased in the NIH/3T3/TCRP1 cells as compared with the control (Figure 1e).

### TCRP1 overexpression induces tumorigenesis of NIH/3T3 cells in nude mice

**Figure 2:**
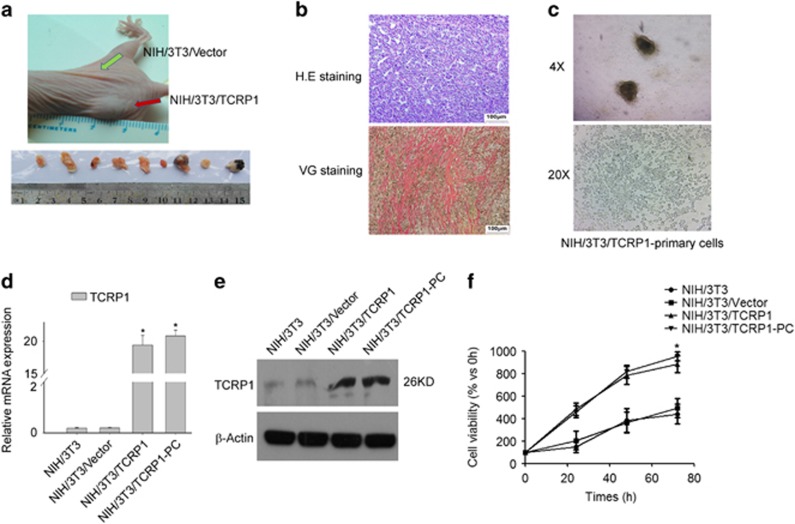
TCRP1 overexpression induces oncogenesis of NIH/3T3 cells in nude mice. NIH/3T3/TCRP1 or NIH/3T3/vector cells injected subcutaneously into the nude mice (*N*=10). Tumor growth was analyzed by measuring tumor length and width. At the end of treatment, tumors were excised (**a**). (**b**) Tumor tissue for pathologic slices with hematoxylin and eosin (H&E) staining and Van Gieson staining individually. (**c**) Tumor tissues of nude mice were primary cultured. (**d**, **e**) Expression of TCRP1 in NIH/3T3/TCRP1 primary culture cells were measured by real-time quantitative PCR (**d**) and western blot (**e**). (**f**) Cell viability was measured by MTS assay. *P <0.05.

NIH/3T3/vector cells and NIH/3T3/TCRP1 cells were inoculated subcutaneously on both sides of the rear back in nude mice. About 3 weeks later, NIH/3T3/TCRP1 cells, but not NIH/3T3/Vector cells, were able to form xenograft (Figure 2a). Hematoxylin and eosin staining showed that the xenograft tissue rich in blood vessels, arranged in disorder, and accompanied with atypical nuclei and mitotic (Figure 2b). We further investigated whether xenograft is fibrosarcoma by adopt special staining (Van Gieson staining). Indeed, the xenograft tissue showed positive Van Gieson staining (Figure 2b), which suggested that the xenograft tissue is fibroblast source. Moreover, we established primary cell lines (NIH/3T3/TCRP1-PC) from xenograft (Figure 2c). Similar to NIH/3T3/TCRP1 cells, NIH/3T3/TCRP1-PC cells showed a marked increase in TCRP1 expression levels (Figures 2d and e), and increase in cell proliferation capacity compared with control cells (Figure 2f).

### TCRP1 directly interacts with PDK1 and activates PDK1/AKT signaling

**Figure 3:**
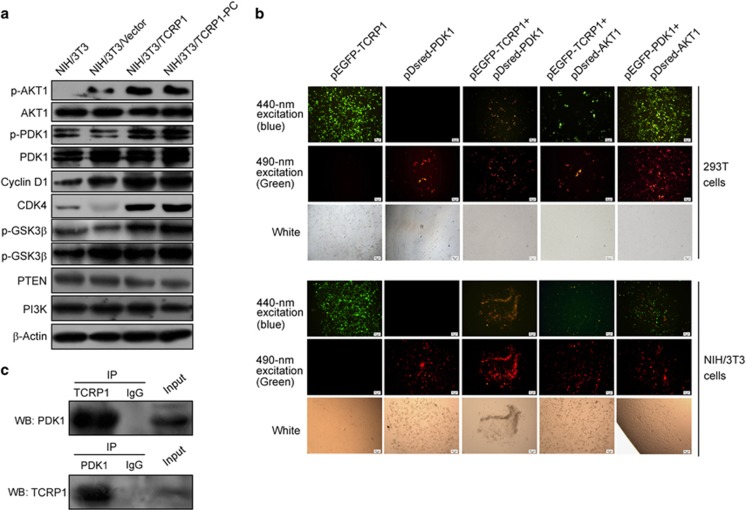
TCRP1 directly interacts with PDK1 and activates PDK1/AKT signaling.(**a**) The effects of TCRP1 on the expression of PI3K/Akt and its downstream molecules. (**b**) 293T and NIH/3T3 cells were co-transfected with EGFP-TCRP1 and pDsred-PDK1, or EGFP-TCRP1 and pDsred-AKT1, the direct interaction between TCRP1 and PDK1, or TCRP1 and AKT1, were determined by forster resonance energy transfer (FRET) assay. Cells co-transfected with EGFP-PDK1 plasmids, and pDsred-AKT1 was used as positive control. (**c**) Total protein extracts of NIH/3T3 cells were subjected to IP using TCRP1 antibody or control IgG, followed by western blotting (WB) with PDK1 antibody (upper panel). Reciprocal IP was done using PDK1 antibody or control IgG, followed by WB with the TCRP1 antibody (lower panel).

Our previous studies suggested that TCRP1 can activate AKT signaling in oral squamous cell carcinoma cells.^8^ Similarly, we found that the phosphoralation levels of AKT (p-AKT) and PDK1 (p-PDK1) was significantly increased in NIH/3T3/TCRP1 cells, as well as NIH/3T3/TCRP1 primary cells (Figure 3a). Furthermore, overexpression of TCRP1 significantly enhanced the protein expression of AKT downstream molecules, including p-GSK3, CKD4 and cyclin D1 (Figure 3a).

PDK1 is known to be activated as a result of the accumulation of PIP3, and PTEN loss and PIK3CA activation both promote the accumulation of PIP3.^11^ However, we found that overexpression of TCRP1 did not affect the expression of PI3K, but modestly decreased PTEN expression (Figure 3a). Interestingly, PIP3 levels in TCRP1-overexpressing NIH/3T3 cells were not significantly different from those seen in control cells (Supplementary Figure S1A), which suggested that TCRP1 activated PDK1 and AKT in a PIP3-independent manner. Thus, we speculated that TCRP1 may have a direct role in activation of PDK1 and AKT. To test this hypothesis, four vectors pEGFP-TCRP1, pEGFP-PDK1, pDsRed-PDK1 and pDsRed-Akt1 were constructed and transfected into NIH/3T3 cells or HEK293 cells, and then analyzed by forster resonance energy transfer assay. As shown in Figure 3b, EGFP energy was transferred to the DsRed fluorescent groups and excites the fluorescent yellow reddish in cells co-transfected with pEGFP-TCRP1 and pDsRed-PDK1, as well as in cells co-transfected with pEGFP-PDK1 and pDsRed-Akt1. However, cells co-transfected with pEGFP-TCRP1 and pDSred-Akt1 did not observe resonance energy transfer (Figure 3b). The results were further confirmed by immunoprecipitation (IP) analyses (Figure 3c), which suggested that TCRP1 can directly interact with the PDK1.

### The PDK1 binding domain of TCRP1 is R93-S107 and T109-A124

**Figure 4:**
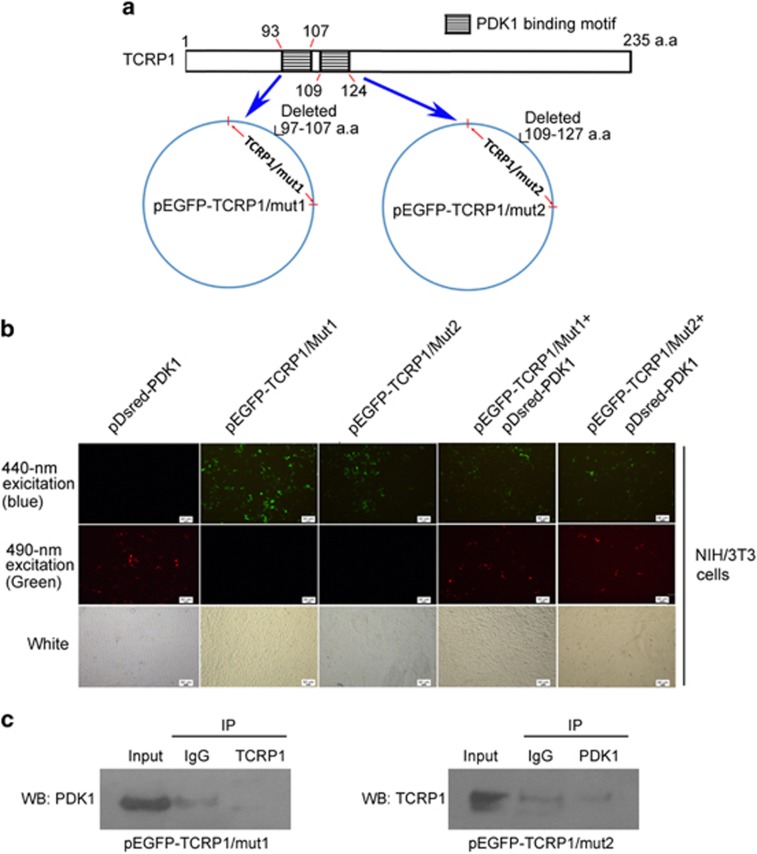
The PDK1-binding domain of TCRP1 is R93-S107 and T109-A124.(**a**) Motif analysis of TCRP1 was processed in Scansite database (http://scansite.mit.edu/), and showed that the 93–107 amino-acid and 109–124 amino-acid sequences in the TCRP1 were PDK1-binding motif (upper panel). Two vectors including the two TCRP1 mutant fragments were constructed named pEGFP-TCRP1/mut1 and pEGFP-TCRP1/mut2, respectively (lower panel). (**b**) NIH/3T3 cells were co-transfected with EGFP-TCRP1/mut1 and pDsred-PDK1, or EGFP-TCRP1/mut2 and pDsred-PDK1, the direct interaction between TCRP1/muts and PDK1 was determined by forster resonance energy transfer (FRET) assay. (**c**) NIH/3T3 cells were transfected with pEGFP-TCRP1/mut1, or pEGFP-TCRP1/mut2, total protein extracts were subjected to IP using TCRP1 antibody or control IgG, followed by WB with PDK1 antibody. Reciprocal IP was done using PDK1 antibody or control IgG, followed by WB with the TCRP1 antibody.

Motif analysis of TCRP1 was processed in Scansite database (http://scansite.mit.edu/), which showed that the 93–107 amino-acid and 109–124 amino-acid sequences in the TCRP1 were PDK1-binding motif (Figure 4a). Then, we constructed two deletion mutant vectors named pEGFP-TCRP1/mut1 and pEGFP-TCRP1/mut2 and transfected them into NIH/3T3 cells, and then analyzed by forster resonance energy transfer assay. Unfortunately, two TCRP1 deletion mutants did not observe resonance energy transfer to PDK1 (Figure 4b), which means TCRP1 cannot bind PDK1 when it lacks 93–107 amino acids or 109–124 amino acids. IP assay also found that TCRP1/muts cannot bind and precipitate PDK1 (Figure 4c). These data suggested both amino acids 93–107 and amino acids 109–124 of TCRP1 are common binding motif to PDK1.

### Inhibition of PDK1 reversed TCRP1-mediated cell transformation of NIH/3T3 cells

**Figure 5:**
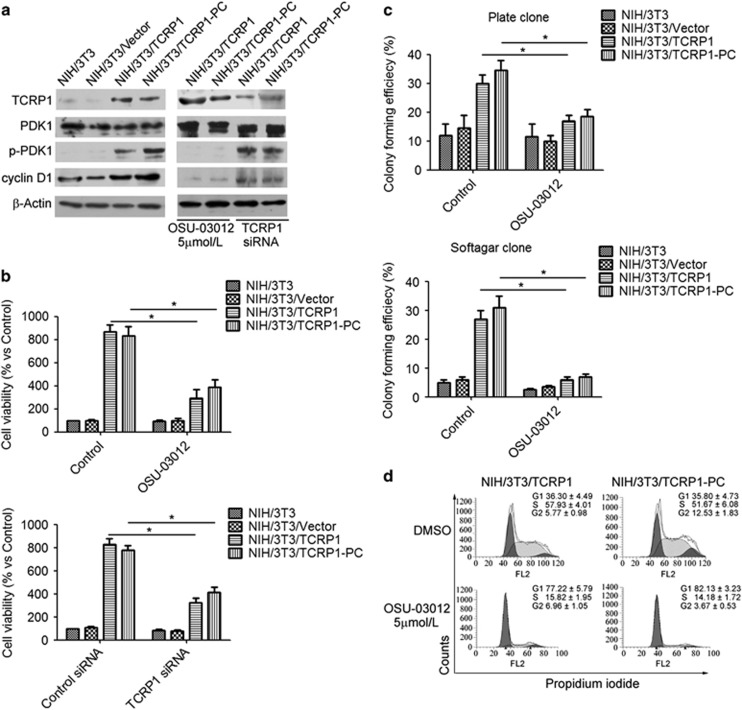
Inhibition of PDK1 reversed TCRP1-mediated cell transformation of NIH/3T3 cells. (**a**, **b**) NIH/3T3/TCRP1 cells or NIH/3T3/TCRP1 primary cells were treated with PDK1 inhibitor OSU-03012, or TCRP1 siRNA, the expression levels of p-PDK1, PDK1 and cyclin D1 were measured by western blotting (**a**); cell viability were measured by MTS assay (**b**). (**c**, **d**) NIH/3T3/TCRP1 cells or NIH/3T3/TCRP1 primary cells were treated with PDK1 inhibitor OSU-03012, clonogenic capacity were measured by plate cloning assay and soft agar cloning assay (**c**). Cells were fixed in ethanol, and stained with propidium iodide, and then DNA contents were determined by flow cytometry (**d**). The percentage of cells in each phase of the cell cycle (G1, S and G2/M) was indicated. Experiments were repeated three times. *P <0.05.

We further investigated whether or not PDK1 is crucial for TCRP1-mediated NIH/3T3 cell transformation. NIH/3T3/TCRP1 and NIH/3T3/TCRP1-PC cells were treated with PDK1 inhibitor OSU-03012, or TCRP1 small interfering RNA (siRNA), respectively. The results showed that treatment of OSU-03012 did not affect the expression of TCRP1. However, treatment of OSU-03012 significantly suppressed the phosphorylation of PDK1 and the expression of cyclin D1 in both NIH/3T3/TCRP1 and NIH/3T3/TCRP1-PC cells (Figure 5a). More importantly, similar to TCRP1 siRNA, OSU-03012 significantly suppressed cell proliferation in both NIH/3T3/TCRP1 and NIH/3T3/TCRP1-PC cells (Figure 5b). To exclude possible off target effect of OSU-03012, NIH/3T3/TCRP1 and NIH/3T3/TCRP1-PC cells were transiently transfected with PDK1 siRNA to block the expression of PDK1 (Supplementary Figure S2A). Consistent with the observation that OSU-03012 suppressed cell proliferation, knockdown of PDK1 also compromised TCRP1-induced cyclin D1 expression (Supplementary Figure S2A) and suppressed cell proliferation in both NIH/3T3/TCRP1 and NIH/3T3/TCRP1-PC cells (Supplementary Figure S2B). Moreover, colony formation assay showed that treatment of OSU-03012 or PDK1 siRNA decreased the colony formation rate of NIH/3T3/TCRP1 cells and NIH/3T3/TCRP1-PC cells (Figure 5c, Supplementary Figure S2C). Furthermore, flow cytometric analysis showed that treatment of OSU-03012, as well as PDK1 siRNA, increased the proportion of G1-phase cells and reduced the proportion of S and G2 phase cells (Figure 5d, Supplementary Figure S2D). Altogether, these data suggested that PDK1 has an important role in TCRP1-mediated cell transformation of NIH/3T3 cells.

### TCRP1 was highly expressed in many tumors and was positively correlated with expression of p-PDK1 and p-AKT

**Figure 6:**
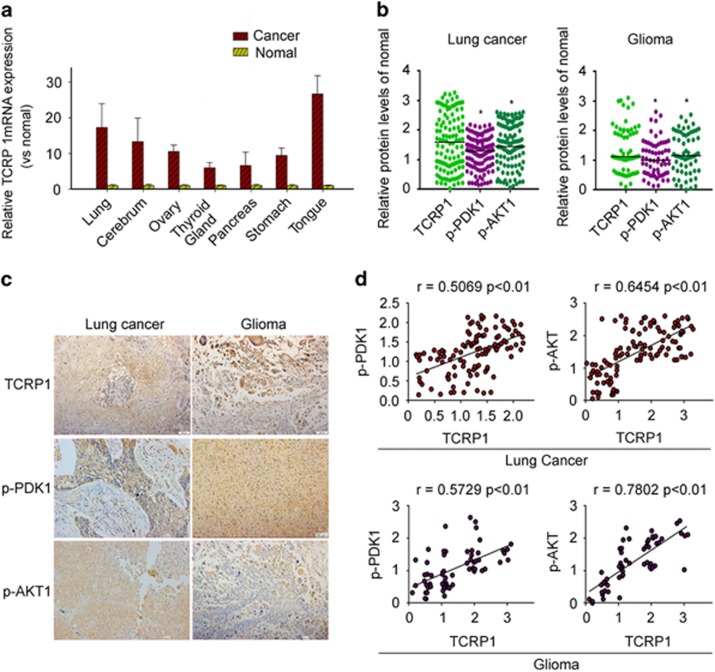
TCRP1 was highly expressed in many tumors and was positively correlated with expression of p-PDK1 and p-AKT. (**a**) Expression of TCRP1 was detected with PCR chip in a variety of tumors and corresponding normal tissue. (**b**) Expression of TCRP1, p-PDK1 and p-AKT1 were detected by immunohistochemistry in lung cancer and glioma. (**c**) Scatter plot showed relative abundance of TCRP1, p-PDK1 and p-AKT1 in lung cancer and glioma; *P <0.05 vs TCRP1. (**d**) The correlation trend of expression of TCRP1, p-PDK1 and p-AKT1 in glioma and lung cancer.

To examine the gene expression levels for TCRP1 in different cancer patient tissue samples, we performed quantitative PCR analysis on TissueScan Cancer and Normal Tissue cDNA Arrays (Origene Company, Rockville, MD, USA), which include 392 cases from different tissues of normal and tumor tissue. We found that expression of TCRP1 in lung cancer, glioma, ovarian cancer, thyroid cancer, nasopharyngeal carcinoma, pancreatic cancer, stomach cancer and tongue carcinoma was significantly higher than that of normal tissue (Figure 6a).

We further analyzed the expression of TCRP1, p-PDK1 and p-AKT1 in primary lung cancer and glioma tissue samples by immunohistochemistry analysis. The results showed that TCRP1, p-PDK1 and p-AKT1 were frequently overexpressed in tumor tissue samples compared with normal tissue samples (Figures 6b and c). More importantly, Spearman correlation analysis showed that the expression levels of TCRP1 has a positive correlation with p-PDK1 (r values=0.5069), or p-AKT1 (r values=0. 6454) in lung cancer tissue (Figure 6d). Similar results were observed in glioma tissue samples, the expression levels of TCRP1 has a positive correlation with p-PDK1 (r values=0.5729), or p-AKT1 (r values=0.7802), respectively (Figure 6d).

## Discussion

Our previous studies suggested that TCRP1 have important roles in chemotherapy and radiotherapy resistance of tongue cancer cells, lung cancer cells and ovarian cancer cells.^8, 9, 10^ In this study, we investigated whether TCRP1 could participate in the oncogenic process. Our results showed that TCRP1 promoted cell transformation and proliferation, which is mostly dependent on activation of PDK1/AKT1 pathway.

We observed that TCRP1 was overexpressed in most of human cancer, including lung cancer, glioma, ovarian cancer, thyroid cancer, nasopharyngeal carcinoma, pancreatic cancer, stomach cancer and tongue carcinoma, which suggested a possible role of TCRP1 in tumor development and progression. Indeed, our results showed that overexpression of TCRP1 promoted cell transformation in NIH/3T3 cells. Similar to most oncogenes, TCRP1 overexpression promoted cell proliferation, and anchorage-independent cell growth, as well as cell cycle progression in NIH/3T3 cells. More importantly, we showed that TCRP1 overexpression induced tumorigenesis of NIH/3T3 cells in nude mice.

In human cancers, PDK1 deregulation in human malignancy can also be caused by gene amplification or abnormal phosphorylation in the cytosol and nucleus, such as in colon cancer and breast cancer.^5, 12^ In this study, we demonstrated that TCRP1 regulates the activity of PDK1, with the evidence of TCRP1 overexpression significantly increased PDK1 phosphoralation levels. PDK1 is thought to be constitutively activated upon elevation of PIP3 owing to the loss of PTEN or gain of PIK3CA activity.^13^ However, our findings suggested that TCRP1-mediated PDK1 phosphorylation is independent on PI3K and PTEN, as TCRP1 did not affect the expression of PI3K, as well as the levels of PIP3. Importantly, we found that TCRP1 can directly bind to PDK1. Therefore, we speculated that TCRP1 may be an adaptor molecule that facilitates PDK1 activation. Homodimerization of PDK1 regulates its activity by maintaining the kinase in an auto-inhibitory conformation.^14^ When PDK1 is in homodimer, it has no phosphorylate activity, while in monomer conformation, it can be self-phosphorylated, and own ability to phosphorylate Akt.^14^ Several adaptor proteins involves in PDK1 activation. For example, the 14-3-3 protein acts as a negative regulator by association with the residues surrounding the Ser-241 residue of PDK1,^15^ whereas serine–threonine kinase receptor-associated protein positively regulates its activity by enhancing its auto-phosphorylation and by stimulating its dissociation from 14-3-3 protein.^16^ In this line, we speculated that TCRP1 binds to PDK1, which may promote PDK1 dissociation from 14-3-3 protein, and promote PDK1 self-phosphorylated, and thus lead to the activation of PDK1 signaling. However, the molecular mechanism by which TCRP1 promotes PDK1 phosphorylation needs to be further elucidated.

Recently, several studies showed that PDK1 signaling has an important role in oncogenic transformation.^17, 18, 19^ Given the central role of PDK1 signaling as an oncogenic driver of tumorigenesis, overexpression of TCRP1 induces NIH/3T3 cell transformation likely through activation of PDK1 signaling. Indeed, our studies demonstrated that inhibition of PDK1 reversed TCRP1-mediated cell transformation of NIH/3T3 cells. AKT is considered the main effector of PDK1 in cancer. PDK1 directly phosphorylates AKT, and then activates AKT signaling.^20^ We found that overexpression of TCRP1 significantly increased AKT phosphorylation, as well as regulated the expression of AKT substrates cyclin D1, and GSK3β. More importantly, we found that the expression of TCRP1 has a positive correlation with p-PDK1, as well as p-AKT1 in human lung cancer and glioma tissue samples. Thus, we suggested that activation PDK1/AKT1 signaling is necessary for TCRP1-induced cell transformation. Recent studies suggested that oncogenic functions of PDK1 may be mediated through the activities of other. For example, it has been recently shown that inhibition of PDK1 has no significant effect on AKT signaling in a PTEN-deficient transgenic tumor mouse model or breast tumor growth,^21, 22^ and oncogenic functions of PDK1 through substrates other than AKT, such as SGK3, mitogen-activated protein kinase or PKCα,^18, 23, 24^ have also been reported. In addition, recent works suggested a novel connection between the PI3K pathway and the MYC oncogene, and identified PDK1–PLK1–MYC signaling as a new oncogenic pathway driving oncogenic transformation.^25^ Therefore, in view of the diversity of PDK1 substrates, whether some other downstream targets of PDK1, such as c-myc, MAPK, involving in TCRP1-mediated cell transformation should be further investigated.

In summary, TCRP1 expression level is increased in human cancer. Moreover, it displays robust oncogenic capacities through activation of PDK1/AKT signaling pathway. These results suggested that TCRP1 represents a promising tumor marker candidate and also a potential target for future therapeutic approaches.

## Materials and methods

### Reagents and antibodies

OSU-03012 was purchased from Selleck Chemicals (Houston, TX, USA). MTS was from Promega (Madison, WI, USA). RPMI-1640 medium and fetal bovine serum was from GIBCO (Invitrogen, Carlsbad, CA, USA). Antibodies against Akt (#9272), phospho-Akt (#4060), PTEN (#9188), PI3K (#4292), GSK3β (#12456), p-GSK3β (#8466), CDK4 (#12790), β-actin (#3700) and cyclin D1 (#2922) were purchased from Cell Signaling Technology (Beverly, MA, USA). Antibody against PDK1 (#sc-7140) was obtained from Santa Cruz Biotechnology (Santa Cruz, CA, USA). Phospho-PDK1 (#ab206340) was obtained from Abcam (Cambridge, MA, USA). TCRP1(#HPA037580) was obtained from Sigma Chemical Co. (St Louis, MO, USA).

### Cell culture and reagents

NIH/3T3 and HEK/293T cell lines were obtained from American Type Culture Collection (Manassas, VA, USA). HEK293FT cells were obtained from Invitrogen. Cell lines were verified using the cell line authentication service at the DKFZ Core Facility by Multiplex human cell authentication in 2011. The cells were cultured in RPMI-1640 containing 10% fetal calf serum (FCS), 100 IU/ml penicillin and 100 μg/ml streptomycin in a humidified incubator of 5% CO_2_ at 37 °C.

### Plasmids and lentiviral vector preparation

The plasmid pEGFP-TCRP1, pEGFP-PDK1, pDsRed-PDK1 and pDsRed-Akt1 were were generated by GeneCopoeia Inc. (Guangzhou, China). To generate lentiviral constructs expressing TCRP1, the PCR products were then cloned into lentiviral vector pLEX/MCS (System Biosciences, Palo Alto, CA, USA) and the sequence confirmed by sequencing (Invitrogen). High titer lentiviruses were generated by transient transfection of 293FT cells with packaging constructs according to the manufacturer's instructions (System Biosciences).

### RNA interference

siRNA for TCRP1 and control siRNAs were purchased from Ruibo Biotech (Guangzhou, China). SignalSilence PDK1 siRNA (#6314) was purchased from Cell Signaling Technology. Cells were transfected with 100 nM siRNA at 60% confluence in reduced-serum RPMI-1640. Lipofectamine 2000 (Invitrogen) was used for transfection following the manufacturer’s protocols.

### Cell viability assay

Cell proliferation was measured in triplicate wells by MTS assay in 96-well plates using the CellTiter 96 Aqueous One Solution Cell Proliferation Assay (Promega), following the manufacturer’s indications.

### Anchorage-dependent and -independent colony formation assays

For plate colony formation assay, the cells in Dulbecco’s modified Eagle’s medium (DMEM) containing 10% FCS were seeded at 1 × 10^3^ cells per well in six-well tissue culture plates. After growing for 10 days at 37 °C, the dishes were stained with crystal violet (Sigma, St Louis, MO, USA)) and colonies of >50 cells were counted under microscope. For soft agar colony formation assay, the cells were suspended in 0.3% agar (Sigma) containing DMEM medium and 10% FCS at a density of 5 × 10^3^ cells/ml. Next, 1 ml of the cell suspension was placed over 1 ml of 0.5% agar containing DMEM medium and 10% FCS in six-well tissue culture plates. After plating, 1 ml of DMEM medium containing 10% FCS was added to the soft agar cultures and replenished every 3 days. Cells were allowed to grow for 12 days and colonies consisting of >50 cells were counted under microscope. All assays were performed three times in triplicate.

### Flow cytometry analysis

The cells (1 × 10^6^ cells) were harvested, washed twice with cold phosphate-buffered saline buffer and fixed with 70% cold ethanol at 4 °C overnight. The fixed cells was then centrifuged, suspended in a buffer (100 mM sodium citrate and 0.1% Triton X-100), and incubated for 15 min at room temperature. The cells were incubated with 10 μg/ml RNase A (Sigma) for 10 min at room temperature, and DNA was stained with 40 μg/ml propidium iodide at 37 °C for 30 min. Samples were immediately analyzed by a FACScan flow cytometry (Becton Dickinson, San Jose, CA, USA). In each analysis, 10 000 events were recorded. The percentage of cells with G1, S and G2 was calculated using Flowjo Analysis Software (Ashland, OR, USA).

### Measurement of PIP3 levels

Cells were collected and washed twice with ice-cold phosphate-buffered saline, and phospholipids were extracted and PIP3 levels were measured by enzyme-linked immunosorbent assay using PIP3 Mass ELISA Kit (#K-2500 s, Echelon Biotechnology, Salt Lake City, UT, USA) according to the manufacturer’s instructions.

### RNA isolation and real-time PCR

Total RNA was isolated from cells using the RNeasy mini kit (Qiagen China Co., Ltd., Shanghai, China) according to the manufacturer's instructions. Complementary DNA synthesis was carried out with the Revert Aid H Minus First Strand cDNA synthesis kit (Fermentas, St Leon-Rot, Germany). The quantitative reverse transcriptase–PCR reactions for target genes were performed using the ABI 7500 Fast sequence detection system (Applied Biosystems, Weiterstadt, Germany), using probes from the Universal Probe Library (Roche Diagnostics, Basel, Switzerland). The housekeeping genes GAPDH were used for normalization of mRNA analysis.

### Western blotting

Cells were lysed in cell lysis buffer containing 1% NP-40, 20 mM Tris-HCl (pH 7.6), 0.15 M NaCl, 3 mM EDTA, 3 mM EGTA, 1 mM phenylmethylsulfonyl fluoride, 20 mg/ml aprotinin and 5 mg/ml leupeptin. Equal amounts of protein were separated by sodium dodecyl sulfate–polyacrylamide gel electrophoresis and transferred to polyvinylidene difluoride membrane (Millipore Corporation, Billerica, MA, USA). After blocking, the blots were probed with the indicated primary antibodies. After washing and incubating with secondary antibodies, the blots were visualized by ECL reagent (Millipore Corporation).

### Immunoprecipitation

Cells were lysed in co-IP buffer (10 mM HEPES (pH 8.0), 300 mM NaCl, 0.1 mM EDTA, 20% glycerol, 0.2% NP-40, protease and phosphatase inhibitors). Lysates were centrifuged and cleared by incubation with 25 μl of Protein A/G gel for 1.5 h at 4 °C. The pre-cleared supernatant was subjected to IP using the indicated first antibodies at 4 °C overnight. Then, the protein complexes were collected by incubation with 30 μl of Protein A/G gel for 2 h at 4 °C. The collected protein complexes were washed 6 × with co-IP buffer and analyzed by western blotting.

### Immunohischemistry

This study was approved by the Ethics Committee of Guangzhou Medical University. Primary tumor specimens were obtained from 102 patients diagnosed with lung cancer, and 85 patients diagnosed with glioma who underwent complete resection in the Affiliated Tumor Hospital of Guangzhou Medical University between 2002 and 2012.

Surgically excised tumor specimens were fixed with 10% neutral formalin, embedded in paraffin and 4-μm-thick sections were prepared. Immunostaining was performed using the avidin–biotin–peroxidase complex method (UltrasensitiveTM, MaiXin, Fuzhou, China). The sections were deparaffinized in xylene, rehydrated with graded alcohol and then boiled in 0.01 m citrate buffer (pH 6.0) for 2 min with an autoclave. Hydrogen peroxide (0.3%) was applied to block endogenous peroxide activity, and the sections were incubated with normal goat serum to reduce nonspecific binding. Tissue sections were incubated with TCRP1 rabbit polyclonal antibody (1:100 dilutions), p-PDK1 antibody (1:200 dilution), p-AKT1 antibody (1:200 dilutions). Staining for antibody was performed at room temperature for 2 h. Biotinylated goat anti-mouse serum IgG was used as a secondary antibody. After washing, the sections were incubated with streptavidin–biotin conjugated with horseradish peroxidase, and the peroxidase reaction was developed with 3,3′-diaminobenzidine tetrahydrochloride.

The intensity of TCRP1, PDK1, AKT1 staining was scored as 0 (no signal), 1 (weak), 2 (moderate) and 3 (marked). Percentage scores were assigned as 1, 1–25% 2, 26–50% 3, 51–75% and 4, 76–100%. The scores of each tumor sample were multiplied to give a final score of 0–12, and the tumors were finally determined as negative (−), score 0; lower expression (+), score ⩽4; moderate expression (++), score 5–8; and high expression (+++), score ⩾9. Tumor sample scored (+) to (+++) were considered positive (overexpression).

### Fluorescence resonance energy transfer

Cells were transiently transfected with EGFP-TCRP1, and/or pDsred-PDK1, and/or pDsred-AKT1 using Lipofectamine 2000 according to the manufacturer’s protocol. Fluorescence imaging was performed at room temperature for 48 h after transfection. Before fluorescence recording, the culture medium was replaced with a solution containing 130 mM NaCl, 5 mM MgCl_2_, 2 mM CaCl_2_, 5 mM HEPES and 1 mM EGTA (pH 7.4). Green fluorescent protein and pDsred images were acquired with excitation at 440 and 490 nm, respectively. Forster resonance energy transfer was recorded by monitoring the quenching of green fluorescent protein during pDsred photobleaching. The images were analyzed with IPLab 4.0 imaging software (Scanalytics, Inc., Rockville, MD, USA).

### Animal studies

All animal work was performed in accordance with protocols approved approved by the Animal Experimentation Ethics Committee of Guangzhou Medical University. Immunodeficient mice, age matched and 4–6 weeks old, were used in assays for tumor growth in a subcutaneous xenograft model. In all, 1 × 10^6^ NIH/3T3/vector cells and 1 × 10^6^ NIH/3T3/TCRP1 cells were inoculated subcutaneous on both sides of the rear back in mice (*N*=9 per group). Tumor growth was analyzed by measuring tumor length (L) and width (W).

### Statistical analysis

Data are presented as mean±s.d.Samples were analyzed by two-tailed unpaired Student's *t*-test, unless otherwise mentioned, and *P*-values <0.05 were considered as being statistically significant.*P*-values <0.05, <0.01 and <0.001 are indicated with one, two and three asterisks, respectively. Data obtained from lung cancer and glioma patient samples were analyzed using GraphPad Prism 5 (GraphPad Software, San Diego, CA, USA). Expression of TCRP1 and *PDK1, AKT1* was correlated using Pearson's correlation.
