# Supplementary figures and images for: TCRP1 promotes NIH/3T3 cell transformation by over-activating PDK1 and AKT1
Source: Oncogenesis. 2017 Apr 24;6(4):e323–. doi: 10.1038/oncsis.2017.18 (PMC5520495; doi:10.1038/oncsis.2017.18)

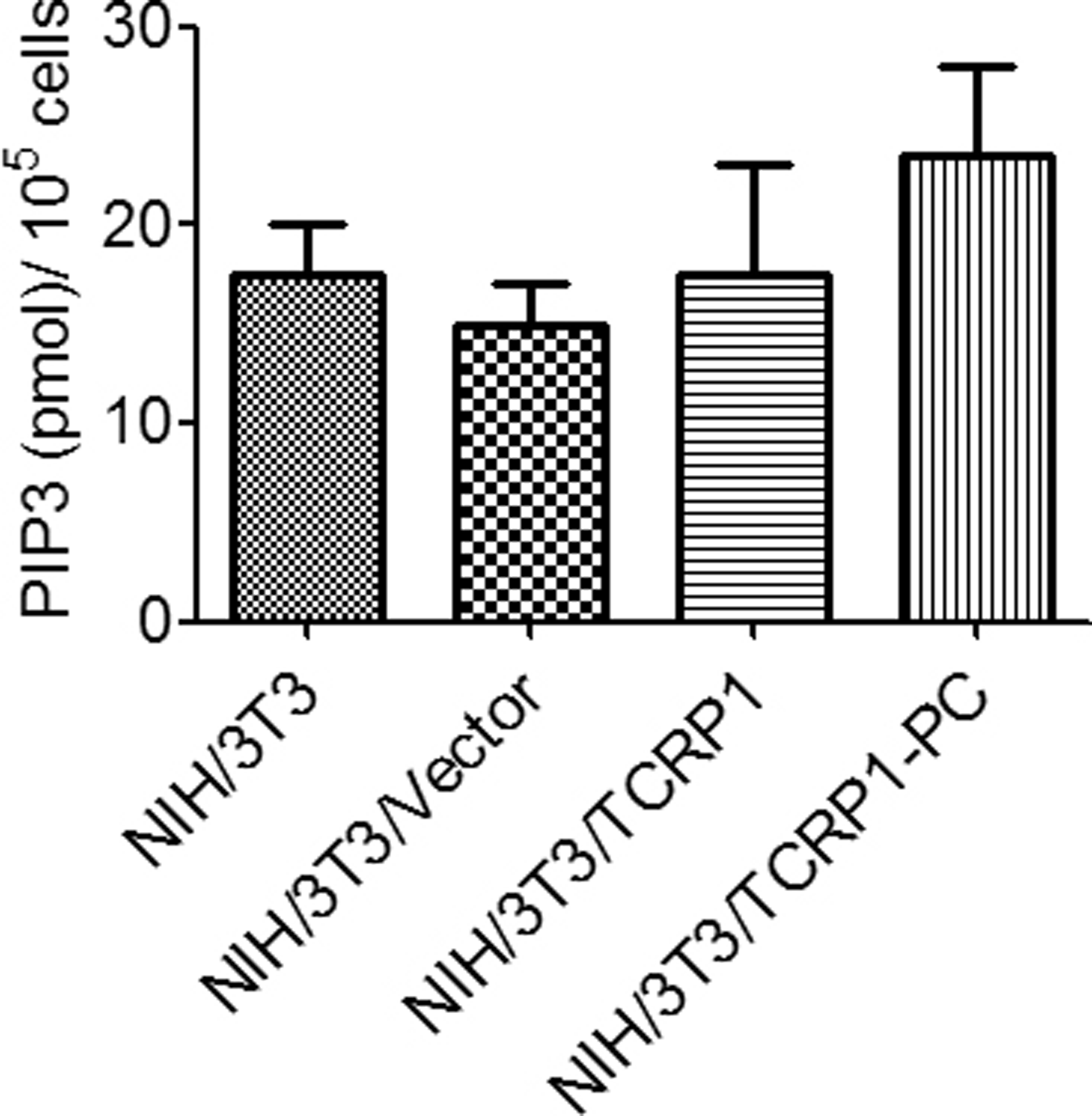

Supplement: Supplementary Figure 1 [file oncsis201718x1.tif]

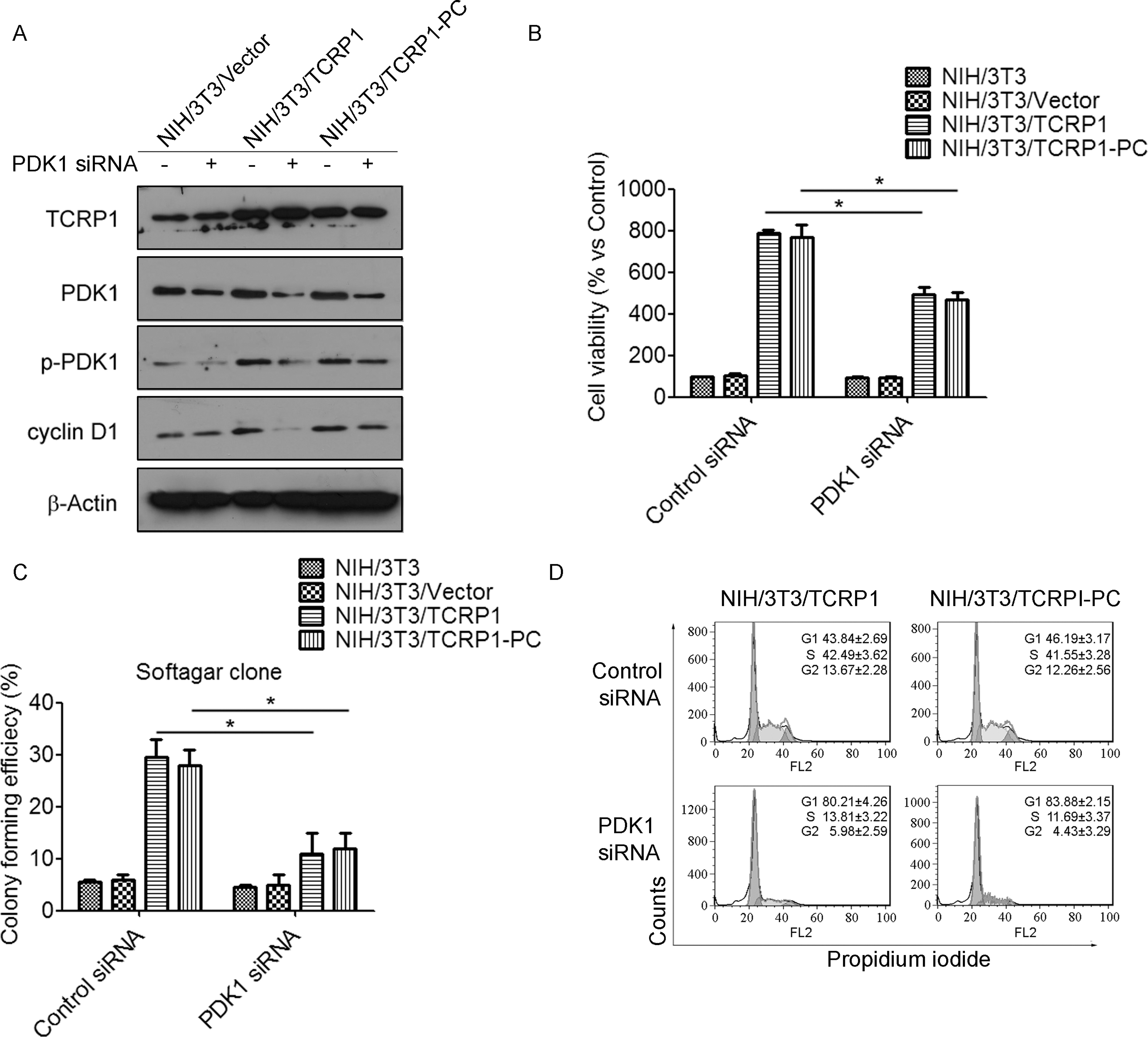

Supplement: Supplementary Figure 2 [file oncsis201718x2.tif]
