# Supplementary material for: TCRP1 promotes NIH/3T3 cell transformation by over-activating PDK1 and AKT1
Source: Oncogenesis. 2017 Apr 24;6(4):e323–. doi: 10.1038/oncsis.2017.18 (PMC5520495; doi:10.1038/oncsis.2017.18)
Supplement: Supplementary Figure Legends [file oncsis201718x3.docx]

**SUPPLEMENTAL FIGURE LEGENDS**

**Supplemental Figure 1.** Effects of TCRP1 overexpression on PIP3 expression levels.

**Supplemental Figure 2.** Knockdown of PDK1 reversed TCRP1-mediated cell transformation of NIH/3T3 cells. NIH/3T3/TCRP1cells or NIH/3T3/TCRP1-primary cells were transfected with PDK1 siRNA, (A) the expression levels of TCRP1, p-PDK1, PDK1, and Cyclin D1 were measured by western blotting; (B) cell viability were measured by MTS assay; (C) clonogenic capacity were measured by soft agar cloning assay; (D) Cells were fixed in ethanol, and stained with propidium iodide, and then DNA contents were determined by flow cytometry, the percentage of cells in each phase of the cell cycle (G1, S and G2/M) was indicated. Experiments were repeated three times. *P <0.05.
